# Real-World Data on Osimertinib-Associated Cardiac Toxicity

**DOI:** 10.3390/jcm14051754

**Published:** 2025-03-05

**Authors:** Abed Agbarya, Ari Raphael, Hadas Gantz Sorotsky, Yakir Rottenberg, Viktor Šebek, Dejan Radonjic, Alexander Yakobson, Johnathan Arnon, Walid Shalata

**Affiliations:** 1 Oncology Department, Bnai Zion Medical Centre, Haifa 31048, Israel; abed.agbarya@b-zion.org.il; 2 Thoracic Cancer Service, Davidoff Cancer Centre, Beilinson Campus, Petah Tikva 49100, Israel; 3 Institute of Oncology, Chaim Sheba Medical Center, Ramat Gan 52600, Israel; 4 School of Medicine, Tel Aviv University, Tel Aviv 69978, Israel; 5 Sharett Institue of Oncology, Hadassah Hebrew University Medical Center, Jerusalem 91200, Israel; 6 Department of Pharmacology, Faculty of Medicine and Dentistry, Palacky University Olomouc, Hnevotinska st. 976/3, 77900 Olomouc, Czech Republic; 7 Faculty of Medicine and Dentistry, Palacky University Olomouc, Hnevotinska st. 976/3, 77900 Olomouc, Czech Republic; 8 The Legacy Heritage Cancer Center and Dr. Larry Norton Institute, Soroka Medical Center, Beer Sheva 84105, Israel; 9 Medical School for International Health, Faculty of Health Sciences, Ben Gurion University of the Negev, Beer Sheva 84105, Israel

**Keywords:** non-small cell lung cancer, osimertinib, cardiac toxicity, real-world data, EGFR mutations

## Abstract

**Background**: Lung cancer is the leading cause of cancer-related deaths globally, with epidermal growth factor receptor (EGFR) mutations present in approximately 17–39% of non-small cell lung cancer (NSCLC) cases. Osimertinib, a third-generation oral EGFR tyrosine kinase inhibitor (EGFR-TKI), has become a cornerstone in the treatment of EGFR-mutated NSCLC. However, the full scope of its potentially life-threatening adverse effects, particularly cardiomyopathy, remains underexplored. **Methods**: This retrospective study was conducted using data from a multi-center registry of NSCLC patients with EGFR mutations treated with first-line osimertinib therapy between December 2018 and April 2024. Osimertinib-related cardiotoxicity was defined as a composite of reduced ejection fraction (EF) and cardiac death. **Results**: The study cohort consisted of 17 patients, and most of the patients had a history of smoking. Cardiac toxicity onset varied from 1 to 28 months following osimertinib initiation, with 70.59% of the patients experiencing symptoms within the first 6 months of treatment. Fourteen patients showed some degree of symptom improvement and EF recovery, although most did not return to baseline EF levels. Comorbidities, including heart failure, hypertension, and dyslipidemia, were prevalent across the cohort. **Conclusions**: While osimertinib remains an effective treatment for EGFR-mutated NSCLC, its associated cardiac toxicity, particularly in patients with pre-existing conditions, presents a significant challenge. Close monitoring, early intervention, and individualized management strategies are critical in mitigating these risks. Patients with mild cardiac toxicity may be suitable for rechallenge, while those with more severe or persistent toxicity should generally be excluded from further osimertinib treatment.

## 1. Introduction

Lung cancer leads the global statistics for cancer-related deaths, surpassing the combined mortality rates of breast, prostate, colorectal, and brain cancers in 2018. Non-small-cell lung cancer (NSCLC) accounts for over 80% of all lung cancer cases and is a major contributor to the high mortality rate associated with this disease. Early stages of NSCLC often present with minimal or no symptoms, causing most patients to seek medical attention only after the cancer has progressed and metastasized. This late diagnosis significantly worsens the outlook for patients [1,2,3,4].

Epidermal growth factor receptor (EGFR) mutations exhibit varying prevalence across different ethnic groups and are present in approximately 17% of Caucasian and 39% of Asian patients with NSCLC, especially non-smokers. Activating mutations in the tyrosine kinase domain of EGFR, the most common being exon 19 deletions and L858R in exon 21, are well-established oncogenic drivers and serve as both biomarkers and a treatment target, particularly for EGFR tyrosine kinase inhibitors (EGFR-TKIs) [5,6,7,8]. Osimertinib is a third-generation oral EGFR-TKI, which, unlike earlier EGFR-TKIs, selectively and irreversibly targets EGFR common mutations and T790M resistance mutations, effectively blocking downstream signaling pathways. Osimertinib’s approval for this indication was initially based on the AURA trial and subsequently on the FLAURA trial, a phase 3 trial that compared osimertinib to first-generation EGFR-TKIs and showed an overall response rate (ORR) of 80%, median progression-free survival (PFS) of 18.9 months, and median overall survival (mOS) of 38.6 months in untreated EGFR-mutated NSCLC patients. The success of this trial led to osimertinib being prioritized in the National Comprehensive Cancer Network (NCCN) guidelines in 2019 as a first-line treatment for metastatic EGFR-mutated NSCLC [8,9,10,11,12]. Furthermore, compared to first-generation EGFR-TKIs, osimertinib was associated with fewer grade 3 adverse events (AE’s), reflecting a better safety profile [13,14].

The FLAURA, AURA trials, and several real-world studies highlighted the AEs associated with osimertinib. The most common side effects, such as diarrhea, rash, paronychia, and stomatitis, are generally manageable and do not necessitate treatment secession. Less frequent, yet clinically significant AEs, including prolonged QT interval and increased liver enzymes, warrant baseline assessment and careful monitoring. The safety profile of osimertinib, combined with its therapeutic benefits, underscores its importance as a first-line treatment option, though individualized patient management remains essential to mitigate potential risks [13,14].

In clinical trials involving osimertinib, transthoracic echocardiogram monitoring identified limited (<5%) incidence of reduced left ventricular ejection fraction (LVEF). However, most affected patients showed no symptoms and were able to continue their osimertinib therapy. Nevertheless, a few case reports and case series have reported symptomatic heart failure (HF) with LVEF linked to osimertinib use [15,16,17,18,19,20,21,22,23]. The extent of this potentially fatal AE is not well understood and broader implications of osimertinib on cardiomyopathy in real-world clinical settings remain unclear, emphasizing the need for further research. In this study, we present real-world data exploring the relationship between osimertinib therapy and cardiomyopathy, including its complications, clinical presentations, and management strategies.

## 2. Materials and Methods

### 2.1. Study Design

**Figure 1:**
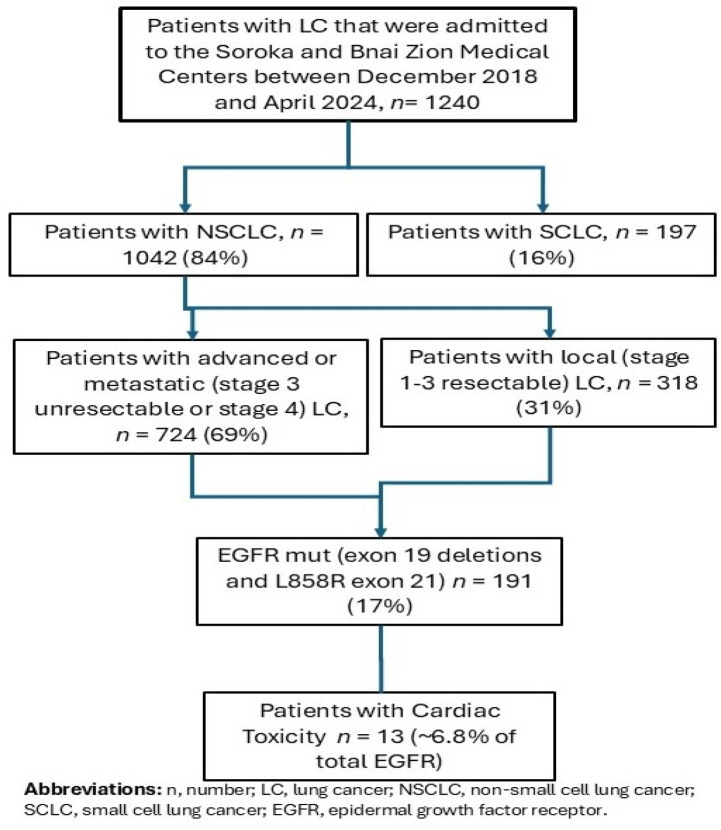
Flow diagram of patients with non-small cell lung cancer treated at different institutions between December 2018 and April 2024.

This retrospective, non-interventional observational study was conducted across multiple institutions in Israel (Soroka Medical Center and Bnai Zion Medical Centre, and individual cases from other medical centers). We retrospectively evaluated our patient databases for all the patients diagnosed with EGFR-mutated NSCLC who received first-line treatment with osimertinib between December 2018 and April 2024. Data collection continued until December 2024. We then evaluated patient medical records and analyzed treatment regimen details, and AE, particularly cardiac AE. Figure 1. illustrates the patient study flow chart.

### 2.2. Clinical Information and Data Collection

#### 2.2.1. Patient Data

Comprehensive patient data were collected, including demographic characteristics, stage at diagnosis, medical and therapeutic histories, performance status measured by Eastern Cooperative Oncology Group (ECOG), treatment regimens and outcomes including start and end dates, last follow-up date, mortality and disease progression dates, overall response rate (ORR), and treatment-related toxicities.

#### 2.2.2. Evaluations, Response Assessment and AE

Response to osimertinib was assessed by respective treating oncologists based on the Related Response Evaluation Criteria in Solid Tumors (RECIST) 1.1. Baseline echocardiograms were performed, or prior echocardiogram results were reviewed. AEs were retrieved from patient medical records and were assessed according to the Common Terminology Criteria for Adverse Events (CTCAE), version 5.0 [24]. To better assess cardiac toxicity, baseline cardiac echograms and ECG prior to osimertinib treatment and periodic echocardiogram ECGs were also recorded to monitor cardiac health. Osimertinib-related cardiomyopathy was defined as a symptomatic or non-symptomatic reduction of 10% in LVEF (with absolute LVEF < 50%) after the start of treatment when other cardiac causes (e.g., IHD) were ruled out [25].

### 2.3. Inclusion Criteria

Patients, 18 or older, with confirmed NSCLC with EGFR mutations (exon 21 or exon 19) validated through tissue or liquid biopsy who received osimertinib monotherapy.

Patients with ECOG scores between 0 and 4 and all relevant comorbidities (e.g., chronic obstructive pulmonary disease (COPD), diabetes, hypertension (HTN), ischemic heart disease (IHD), heart failure (HF), or obesity) were included. We excluded patients who received osimertinib in combination with chemotherapy or in advanced lines.

### 2.4. Data and Statistical Analysis

Descriptive statistics were used to analyze all the parameters in this study, including percentages, ranges, means, medians, and interquartile ranges (IQRs). The study parameters included both numerical data (e.g., age, months from the start of treatment to heart failure, and EF data) and binary data (e.g., smoking status at diagnosis, heart failure, hypertension, diabetes, COPD, ischemic heart disease, obesity, and dyslipidemia, with Yes/No or 1/0 responses). Descriptive statistics were also applied to summarize treatment outcome measures, including adverse events (AEs), expressed as percentages (%); changes in EF at baseline, during, and after osimertinib-related heart failure; and the distribution of these measures across the study population.

## 3. Results

### 3.1. Patient Characteristics

Between December 2018 and April 2024, we identified 191 patients with EGFR-mutated NSCLC treated with first-line osimertinib. Of them, 17 patients (Table 1), with a mean age of 72.7 years (range 60–83) suffered from osimertinib-related cardiomyopathy. The cohort consisted of 10 females (58.82%) and 7 males (41.18%). Most patients were diagnosed at an advanced stage, 13 patients (76.47%) with stage 4 disease, 2 patients (11.76%) with stage 2 disease, and 2 patients (11.76%) with stage 3 disease. Concerning smoking status, 6 patients (35.29%) were either former or current smokers, whereas 11 patients (64.71%) had never smoked. EXON 19 deletions were observed in 10 patients (58.82%) and 7 patients (41.18%) had the EXON 21 (L858R) mutation.

Comorbidities were prevalent in the study population. HF at diagnosis was present in 9 patients (52.94%), HTN was observed in 10 patients (58.82%), and diabetes was present in 4 patients (23.53%). IHD and obesity were each present in six patients (35.29%). Further patient characteristics and comorbidities are detailed in Table 1.

### 3.2. Treatment Outcomes and Cardiac AE

In response to osimertinib treatment (Table 2), 12 patients (70.59%) exhibited a partial response, while 4 patients (23.53%) achieved a complete response. All cases of osimertinib-related cardiotoxicity in this cohort were diagnosed as a result of symptoms and not serial cardiac echograms. The most common symptoms leading to investigations of suspected cardiomyopathy were dyspnea (9 patients, 52.94%), fatigue (8 patients, 47.06%), cough (7 patients, 41.18%), and chest pain (5 patients, 29.41%).

The baseline LVEF varied among patients, ranging from 30% to 65%. LVEF decreased significantly during the development of osimertinib-related HF, with an average change of 21% (range 10–55%) with the lowest LVEF observed during treatment falling as low as 10%. For example, one patient with a baseline EF of 65% experienced a reduction to 35% during heart failure, followed by a recovery to 50% after discontinuing osimertinib and initiating HF. Similarly, another patient with a baseline EF of 55% during heart failure showed improvement to 45% after starting heart failure management. The onset of cardiac toxicity after starting osimertinib was varied and ranged from 1 to 28 months, with most patients (70.59%) experiencing cardiac toxicity within the first 6 months of treatment, yet 4 patients displaying symptoms more than two years after starting treatment; notably, none of the 17 patients suffered from additional prolonged QT, which may have attributed to the observed HF.

### 3.3. Management of Osimertinib Cardiac Toxicity

Once diagnosed, osimertinib treatment was withdrawn for all the patients. All 17 patients were referred to a cardiologist, and all of our patients were treated with a combination of angiotensin-converting enzyme (ACE) inhibitors, beta-blockers, and diuretic spironolactone to address the reduction in LVEF. For patients who did not experience improvement in LVEF after nearly a year of treatment, the ACE inhibitors were switched to an angiotensin receptor blocker (Entresto). Diagnostic catheterization was performed in eight patients (47.06%) during periods of significant HF. In terms of recovery, 14 patients (64.71%) showed some degree of symptom improvement and EF recovery after osimertinib was stopped and HF medication was administered, although most did not return to their baseline EF levels. Importantly, one patient died as a result of osimertinib cardiac toxicity.

**Figure 2:**
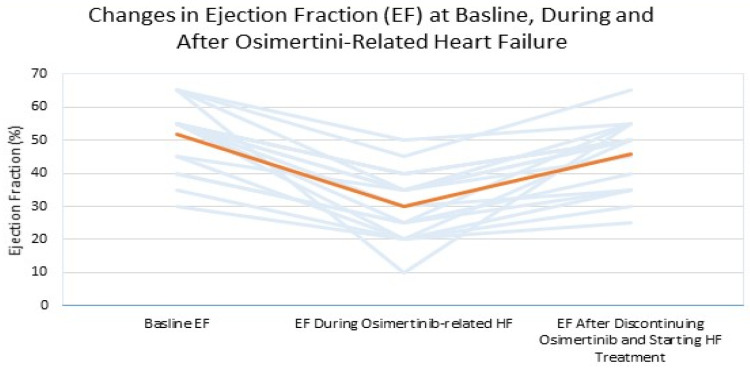
Changes in LVEF at baseline, during osimerinib-related HF, and after recovery (blue lines explaining for each patient, orange line is the median).

Rechallenge with osimertinib was attempted in 11 patients (64.71%). Six patients (35.29%) did not undergo rechallenge due to severe toxicity or fragile health status. Among those who were rechallenged, seven patients (41.18%) successfully resumed osimertinib after receiving treatment for HF and continued to show signs of cardiac recovery. Of them, four patients (23.53%) had their osimertinib dose reduced to 40 mg due to concerns about the potential recurrence of cardiac toxicity (Figure 2).

### 3.4. Other Osimertinib AEs

Osimertinib was associated with a range of AEs in our patient cohort, excluding cardiac toxicity (Table 3). The most common was fatigue, affecting 14 patients (82.35%) in grade 1 and 3 patients (17.65%) in grade 2. Rash, paronychia, and anemia were also frequently observed, with three patients (17.65%) experiencing grade 1 rash and paronychia, and three patients (17.65%) experiencing grade 1 anemia. Additionally, anemia was noted in two patients (11.76%) at grade 2.

Other relatively common AEs included thrombocytopenia, which affected two patients (11.76%) at grade 1 and one patient (5.88%) at grade 2, as well as nausea, reported in two patients (11.76%) at grade 1. Both neutropenia and diarrhea were observed in one patient (5.88%) at grade 1 and grade 2. Less frequently, abdominal pain was reported in one patient (5.88%) at grade 1. No grades 3–4 were reported.

## 4. Discussion

In this study, we report osimertinib-related cardiac HF incidence and medical management from real-world data from several referral centers in Israel over the past 5 years. While osimertinib has proven effective for most patients with EGFR-mutated NSCLC in both clinical trials and real-world practice, cardiac toxicity remains a significant concern, particularly for those at higher risk, and requires careful monitoring and management [7,26,27].

Major clinical trials, including the FLAURA and AURA trials and real-world studies involvingo, found a 4–5% incidence of reduced LVEF. In the FDA Adverse Event Reporting System (FAERS), 6.1% of all osimertinib-related AEs were cardiac in nature, and the majority prolonged QTc. Notably, no fatal cardiac Aes related to osimertinib were reported in clinical trials. In our study, nearly 6.8% of the patients experienced osimertinib-related HF, with one patient succumbing to cardiac toxicity. Furthermore, in the FLAURA and AURA trials, most cases of HF were a result of the serial monitoring of cardiac echograms (which were available for over 90% of the patients), while in our cohort, HF was diagnosed as a result of new symptoms, suggesting that this AE is even more prevalent in our population [13,14,26,27]. It is important to recognize that the higher risk of HF in EGFR-mutated NSCLC patients may stem from a combination of factors, including older age, pre-existing cardiac conditions, and other comorbidities commonly observed in real-world patients. The addition of osimertinib to these patients’ treatment regimens may further increase the risk of developing heart failure. It should be noted that this higher incidence is likely due to the greater flexibility in osimertinib treatment in real-world settings compared to the more controlled environment of clinical trials. It was noted that the patients in our cohort were older and frailer than those typically enrolled in clinical trials and had more complex medical histories. Many of our patients had pre-existing conditions known to predispose them to HF, such as dyslipidemia (64.71%), hypertension (58.82%), heart failure (52.94%), ischemic heart disease (35.29%), obesity (35.29%), previous heart catheterization (29.41%), and diabetes (23.53%). These factors, combined with osimertinib treatment, may contribute to an increased risk of serious cardiac adverse events.

Regarding recovery from decreases in LVEF, the FLAURA study reported that eight (3.1%) patients in the osimertinib arm and three (1.2%) patients in the comparator EGFR-TKI arm experienced a decrease in LVEF from baseline by ≥10 percentage points, resulting in an absolute LVEF value of <50%. All eight patients who had a decrease in LVEF while on osimertinib recovered while continuing on the full dose of osimertinib, with no cardiac symptoms observed. Additionally, in the AURA study, adverse events related to osimertinib included LVEF decrease in six (2.2%) patients and cardiac failure in three (1.1%) patients [7,16,28].

In a pooled analysis of all the osimertinib-treated patients across clinical studies, 908 patients had both baseline and at least one follow-up LVEF assessment. A decrease in LVEF of ≥10 percentage points, with an absolute LVEF value < 50%, was observed in 35 (3.9%) patients. Furthermore, recovery outcomes were reported, with 20% of the patients recovering, 10% in the process of recovery, and 2% recovering with sequelae [7,16,28].

Importantly, of the 17 patients included in this cohort, 14 were able to recover after secession of osimertinib with the aid of HF medication, and in 11 cases, osimertinib was safely rechallenged. Considering the clinical benefit of osimertinib and limited second-line options, this strategy illustrates the importance of personalized patient management and the importance of close cardiac monitoring and cardioprotective HF medication which allowed to maximize the utility of osimertinib in this cohort.

Although no fatalities have been reported due to osimertinib-related cardiac toxicity, several safety warnings are associated with its use, particularly concerning cardiac AEs. In the United States, patients with cardiac risk factors or those at increased risk for QTc prolongation—such as those taking QTc-prolonging drugs—are advised to undergo regular cardiac monitoring. If significant cardiac events occur, osimertinib may need to be withheld, dose-adjusted, or discontinued [29,30].

The mechanism of osimertinib-related HF is not well understood. Some preclinical data suggest that osimertinib can cause direct cardiomyocyte death [31]. However, the varied range of onset and the successful recovery and rechallenge witnessed in our cohort and others [14,15,16,23] suggest a subtler non-dose-dependent and reversible insult. Osimertinib and its metabolite are known to inhibit HER2, which activates the survival pathway of the myocardium through the dimerization of the HER4 receptor binding to neuregulin. Additionally, osimertinib blocks the RAS/RAF pathways, which are needed to stabilize myofibril structure and inhibit apoptosis [32]. Therefore, osimertinib is thought to share a similar mechanism of cardiac damage as anti-HER2 agents, such as trastuzumab, suggesting that the predisposition and transient effect for osimertinib-related HF might be similar.

While age and a history of HF were previously found to be associated with osimertinib-related HF, further research is needed to assess whether increased susceptibility to cardiac AEs from osimertinib is linked to specific risk factors for heart disease. Additionally, it is not clear if baseline and serial cardiac echograms are warranted and could have prevented the mortality reported in this cohort. Finally, more post hoc studies should explore the precise mechanisms behind osimertinib-associated cardiac toxicity and its potential complications to identify patients at risk.

Echocardiography is one of the most effective methods for follow-up, as it is readily available and more cost-effective compared to other cardiac imaging tests. Its feasibility, reproducibility, and accuracy have been widely demonstrated, with myocardial strain measurements across various chambers now incorporated into diagnostic algorithms and guidelines for different pathologies [33]. Even though none of the patients had direct cardiac involvement from lung carcinoma. However, direct cardiac involvement by the neoplastic process is possible and can increase the risk of heart failure and other complications. Therefore, we recommend following up with echocardiography every 3 to 4 months regardless of whether there is direct cardiac involvement.

It is important to mention that for EGFR-mutated NSCLC patients where rechallenge is not an option, reducing the dose to 40 mg daily instead of 80 mg is one potential strategy. However, it was more commonly reported that patients were switched to erlotinib, which was implemented in the majority of cases, with a few instances where a switch to afatinib was considered [34,35,36,37].

Our study observed a lower incidence of both grade 1–2 AEs, with no grade ≥ 3 AEs reported. In patients receiving osimertinib, the most reported AEs, as assessed by investigators, included rashes and acne, diarrhea, dry skin, paronychia, stomatitis, pruritus, decreased appetite, and elevated liver enzymes. When comparing these AEs with our real-world data, we observed that the majority of AEs were dermatologic in nature. Additionally, hematological AEs, such as anemia and thrombocytopenia, were observed, with these events also occurring predominantly in grades 1–2. This low trend is likely attributable to the extensive clinical experience of our treatment team and their heightened vigilance in monitoring patients. The prompt identification and effective management of AEs not only benefited patients but also likely contributed to improved overall treatment outcomes and enhanced quality of life.

Our study has several limitations that should be taken into account. These include the small sample size, which is partly due to the rarity of the AE under investigation; the retrospective design; and the fact that all the cases included were a result of symptoms and not cardiac monitoring, which limits us from understanding the exact prevalence of this AE. Additionally, the patient databases utilized were limited to those under the jurisdiction of Oncology Departments, which means that some patient data may have been overlooked if those patients were recorded in alternative department databases.

## 5. Conclusions

While osimertinib has proven effective for most patients with EGFR-mutated NSCLC, osimertinib-related HF remains a significant concern and might be more prevalent in real-world settings due to the frailer, older patient population and particularly for those at higher risk. Close monitoring, including echocardiogram and the use of HF medications, is essential for managing this AE as most patients are likely to recover and can be considered for rechallenge with osimertinib, maximizing its therapeutic potential.

## Figures and Tables

**Table 1:** The baseline characteristics of the study population (n = 17).

Characteristics		Mean (Range)
Age (years)		72.7 (60–83)
	Female	73 (60–83)
	Male	72.8 (62–80)
Gender		Frequencies (percentage)
	Female	10 (58.82)
	Male	7 (41.18)
Stage at Diagnosis		
	2A	1 (5.88)
	2B	1 (5.88)
	3B	2 (11.76)
	4	13 (76.47)
Smoking status (at diagnosis)		
	Former or Current	6 (35.29)
	Never	11 (64.71)
Histology		
	Adenocarcinoma	16 (100)
EGFR mutation types		
	EXON 19 deletions	10 (58.82)
	EXON 21 (L858R point mutation)	7 (41.18)
Comorbidities at diagnosis		
Previously heart catheterization		
	Yes	5 (29.41)
	No	12 (70.59)
Heart failure		
	Yes	9 (52.94)
	No	8 (47.06)
Hypertension		
	Yes	10 (58.82)
	No	7 (41.18)
Diabetes		
	Yes	4 (23.53)
	No	13 (76.47)
COPD		
	Yes	1 (5.88)
	No	16 (94.12)
IHD		
	Yes	6 (35.29)
	No	11 (64.71)
Obesity		
	Yes	6 (35.29)
	No	11 (64.71)
Dyslipidaemia		
	Yes	11 (64.71)
	No	6 (35.29)

Abbreviations: COPD, chronic obstructive pulmonary disease; IHD, ischemic heart disease; EGFR, epidermal growth factor receptor.

**Table 2:** Response to osimertinib and cardiac toxicity outcomes.

Response	Symptoms Lead to Investigations	Background EF	EF During HF	EF After Discontinuing Tagrisso and Starting HF Treatment	Medications Due to HF	Catheterization During Insufficiency	Recovery	Months Since Starting Treatment to HF	Rechallenge of Osimertinib
PR	Dyspnea and cough	65	35	50	Yes	Yes	Yes	11	No
PR	Dyspnea and cough	55	30	35	Yes	Yes	No	26	No
PR	Angina and cough	35	20	35	Yes	Yes	Yes	9	No
PR	Cough, chest pain, fatigue, and pleural effusion	55	35	45	Yes	No	Yes	5	Yes
PR	Chest pain and cough	65	45	65	Yes	No	Yes	29	Yes
PR	Dyspnea, cough, and fatigue	55	40	50	Yes	Yes	Yes	6	Yes
PR	Atrial fibrillation and fatigue	30	20	25	Yes	No	Yes	1	Yes
PR	Pericardial effusion, peripheral edema, and fatigue	55	35	50	Yes	Yes	Yes	8	Yes, 40 mg
PR	Chest pain and cough	65	50	55	Yes	Yes	Yes	5	Yes
CR	Dyspnea and orthopnea	55	20	50	Yes	Yes	Yes	6	Yes, 40 mg
PR	Dyspnea and peripheral edema	45	20	30	Yes	No (fragile state ECOG 3)	Yes	26	Yes, 40 mg
PR	Dyspnea	45	35	55	Yes	No	Yes	4	Yes, 40 mg
PR	Pericardial effusion, dyspnea, peripheral edema, and fatigue	40	25	35	Yes	No (fragile state ECOG 4)	No	3	No
CR	Chest pain and dyspnea	55	25	55	Yes	Yes	Yes	6	Yes
CR	Chest pain and fatigue	55	40	50	Yes	No	Yes	7	Yes
CR	Peripheral edema and fatigue	40	25	40	Yes	No	No	10	No
CR	Pleural effusion, dyspnea, and fatigue	65	10	55	Yes	No (fragile state ECOG 4)	Yes	28	No

Abbreviations: EF, ejection fraction; HF, heart failure; PR, partial response; CR, complete response.

**Table 3:** Adverse events associated with osimertinib in the patient cohort (excluding cardiac toxicity).

Adverse Event	Grade 1 (%)	Grade 2 (%)
Fatigue	14 (82.35)	3 (17.65)
Rash	3 (17.65)	1 (5.88)
Paronychia	3 (17.65)	1 (5.88)
Anemia	3 (17.65)	2 (11.76)
Thrombocytopenia	2 (11.76)	1 (5.88)
Nausea	2 (11.76)	0
Neutropenia	1 (5.88)	1 (5.88)
Diarrhea	1 (5.88)	1 (5.88)
Abdominal pain	1 (5.88)	0
Pancreatitis	0	1 (5.88)
Renal failure	0	1 (5.88)

## Data Availability

The data either resides within the article itself or can be obtained from the authors upon making a reasonable request.
